# Multi-disciplinary cooperation for the micro-elimination of hepatitis C in China: a hospital-based experience

**DOI:** 10.1186/s12876-023-03016-7

**Published:** 2023-11-11

**Authors:** Lingling Zheng, Xiaoli Zhang, Yuxia Nian, Wenjuan Zhou, Dan Li, Yong Wu

**Affiliations:** 1 Department of Prevention and Healthcare, Fujian Medical University Union Hospital, 29 Xinquan Road, Gulou, Fuzhou, Fujian 350001 P. R. China; 2 Department of Laboratory Medicine, Fujian Medical University Union Hospital, 29 Xinquan Road, Gulou, Fuzhou, Fujian 350001 P. R. China; 3 Department of Gastroenterology, Fujian Medical University Union Hospital, 29 Xinquan Road, Gulou, Fuzhou, Fujian 350001 P. R. China; 4 Department of Medical Affair, Fujian Medical University Union Hospital, 29 Xinquan Road, Gulou, Fuzhou, Fujian 350001 P. R. China

**Keywords:** Hepatitis C virus, Hospital, Micro-elimination, Multi-disciplinary cooperation, Patient follow-up, Health policy

## Abstract

**Background:**

Hepatitis C virus (HCV) infection is one of the main causes of liver cancer and imposes an enormous social and economic burden. The blood-borne virus screening policy for preventing iatrogenic infections renders hospitals important for identifying individuals infected with hepatitis C. Therefore, we aimed to investigate the establishment of a multi-disciplinary cooperation model in medical institutions to leverage the screening results of patients with hepatitis C. Our objective is to ensure that patients receive timely and effective diagnosis and treatment, thereby enabling the elimination of hepatitis C by 2030.

**Method:**

A multi-disciplinary cooperation model was established in October 2021. This retrospective study was based on the establishment of antibody-positive and HCV RNA-positive patient databases. A Chi-square test was used to compare the HCV RNA confirmation rate in anti-HCV-positive patients, as well as the hepatitis C diagnosis rate and treatment rate in RNA-positive patients before and after the multi-disciplinary cooperation. A multivariable logistic regression was used to analyse the factors affecting the treatment of patients with hepatitis C. In addition, we examined changes in the level of hepatitis C knowledge among medical staff.

**Results:**

After the implementation of the multi-disciplinary cooperation model, the RNA confirmation rate of hepatitis C antibody-positive patients increased from 36.426% to 88.737%, the diagnostic accuracy rate of RNA-positive patients increased from 67.456% to 98.113%, and the treatment rate of patients with hepatitis C increased from 12.426% to 58.491%. Significant improvements were observed among the clinicians regarding their ability to understand the characteristics of hepatitis C (93.711% vs. 58.861%), identify people at high risk (94.340% vs. 53.797%), manage patients with hepatitis C after diagnosis (88.679% vs. 67.089%), and effectively treat hepatitis C (84.277% vs. 51.899%). Multi-disciplinary cooperation in medical institutions was the most important factor for patients to undergo HCV treatment (odds ratio: 0.024, 95% confidence interval: 0.007–0.074).

**Conclusions:**

This study showed that the use of a multi-disciplinary cooperation model to utilise the results of HCV antibody screening fully in patients through further tracking, referral, and treatment may facilitate the detection and treatment of patients with hepatitis C and accelerate the elimination of HCV in China.

## Background

Hepatitis C is an inflammatory liver disease caused by the blood-borne hepatitis C virus (HCV). It is primarily transmitted through unsafe injections, unscreened blood transfusions, and injection drug use [1]. Chronic infection with hepatitis C virus can lead to liver cirrhosis and cancer [2, 3]. The introduction of direct-acting antiviral agents (DAAs) has revolutionised hepatitis C treatment, with most patients achieving a high sustained virologic response (SVR) rate (≥ 90%) following 8–12 weeks of DAA treatment [4, 5]. In line with these treatment success rates, the World Health Organization (WHO) in their 2016 Global Health Sector Strategy has set the following goals for HCV elimination by 2030: 90% and 65% reductions in the number of new HCV infections and HCV-related mortality, respectively, and 90% and 80% diagnosis and treatment rates of HCV infection, respectively [6, 7].

According to the data published by Polaris Observatory HCV Collaborators, there were an estimated 9.487 million HCV-infected cases in China in 2020 [8]. There were regional variations in anti-HCV-positivity rates, with higher rates in the north (0.53%) compared with those in the south (0.29%) [9]. Approximately 4.56 million patients require treatment for chronic hepatitis C, as delayed treatment can result in liver cirrhosis or cancer [10]. Previously, China had announced its response to the WHO’s initiative [11]. However, the coronavirus disease 2019 (COVID-19) pandemic has impeded progress towards hepatitis C elimination [12, 13]. Widespread screening spanning the entire population is currently not feasible owing to resource constraints.

In 2017, the International Liver Foundation of the European Association for the Study of the Liver published a pragmatic strategy using micro-elimination. This approach involves a focus on targeted populations, such as incarcerated individuals [14], patients with AIDS [15], patients undergoing haemodialysis [16], those at high risk of HCV infection, or those residing in specific regions [17, 18]. In hospitals, the target population may primarily include those with HCV infections identified through blood-borne virus screening policies, particularly among patients undergoing invasive procedures, transfusions, and surgeries [19]. However, the effective analysis of screening results for early detection and treatment requires specialist knowledge. Consequently, non-specialised physicians face challenges when diagnosing and treating hepatitis C. Screening results are mainly used to warrant preventive measures to avoid nosocomial infections, while consultations and referrals, which lead to patient diagnosis and treatment, are evaded [19]. To improve upon this, hospital-based micro-elimination strategies should focus more attention to current hepatitis C diagnoses and treatments rather than identifying patients previously diagnosed as HCV-seropositive [19, 20].

At the Fujian Medical University Union Hospital, we implemented a multi-disciplinary cooperation model to achieve in-hospital micro-elimination of hepatitis C. We present the details of our model in this paper, aiming to contribute towards meeting the WHO Global Health Sector Strategy targets for HCV elimination.

## Methods

### Establishment of the hepatitis C micro-elimination management system

In August 2021, a hepatitis C multi-disciplinary cooperation group was formed at the hospital, led by the hospital leader. This group integrated various medical resources from departments such as medical affairs, prevention and healthcare, hospital-acquired infection control, infectious diseases, gastroenterology, clinical laboratory, information management, and pharmacy.

**Fig. 1:**
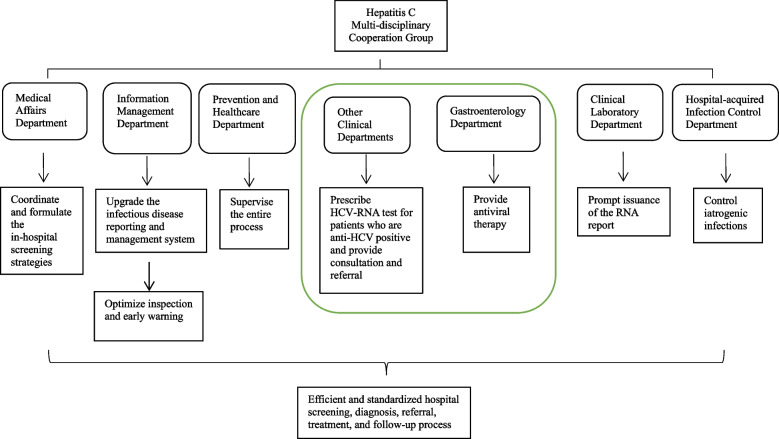
Specific division of responsibilities of the hepatitis C multi-disciplinary cooperation group

**Fig. 2:**
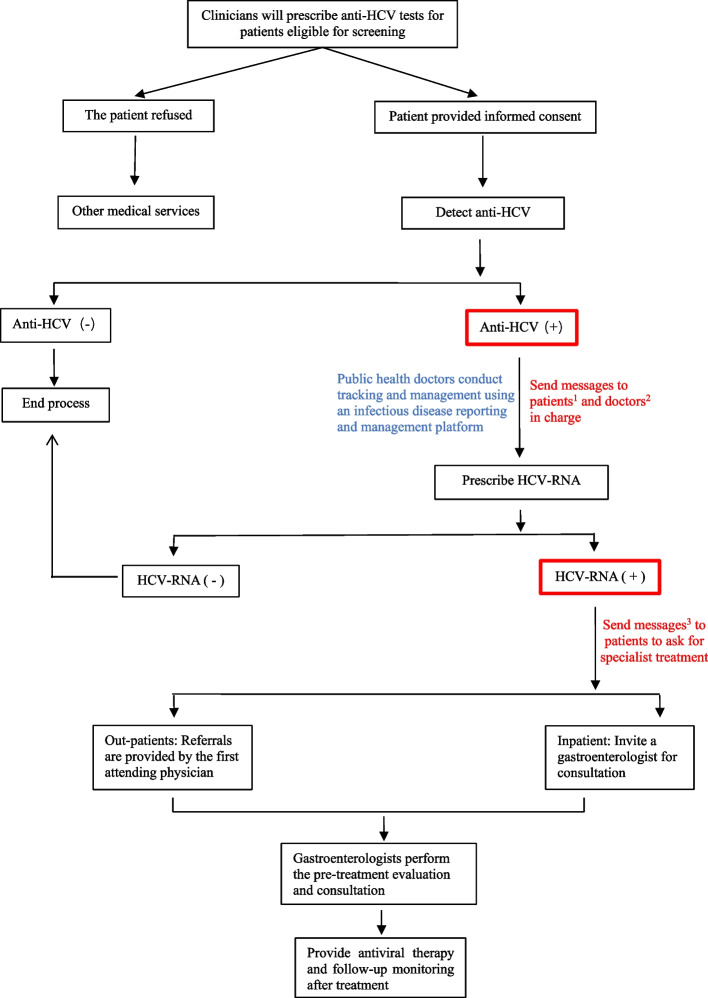
Screening, diagnosis, and referral process of hepatitis C cases in the hospital. ^1^Hello, XXX. Your hepatitis C antibody test result at our hospital was positive. For your and your family’s health, please visit the hepatitis C clinic of the Department of Gastroenterology for a follow-up diagnosis. ^2^Dr. XXX, the hepatitis C virus antibody test result for your patient XXX (patient ID XXXXXX) is positive; please ask the patient to undergo the hepatitis C virus RNA test to confirm the diagnosis of hepatitis C. If a patient is diagnosed with hepatitis C, please refer the patient to the hepatitis C clinic of the Department of Gastroenterology. ^3^Hello, XXX. Your hepatitis C RNA test result at our hospital was positive. For your and your family’s health, please visit the hepatitis C clinic of the Department of Gastroenterology for treatment

The medical affairs department formulated and improved testing strategies, including implementing hepatitis C examination regulations for patients undergoing surgery, hospitalisation, haemodialysis, invasive diagnosis, and treatment. The hospital-acquired infection control department focused on preventing iatrogenic infections. The clinical laboratory department implemented a system to remind clinicians to order HCV RNA tests for patients with positive anti-HCV test results and added notes on reports with positive HCV RNA results to visit the hepatitis C clinic in the gastroenterology department. The information management office upgraded the infectious disease reporting and management system, enabling automatic Short Message Service (SMS) notifications to doctors in charge of patients with positive anti-HCV or HCV RNA results regarding follow-up diagnosis and treatment. Public health doctors in the prevention and healthcare department focused on quality control, while monitoring and managing patients with hepatitis C through an infectious disease information reporting platform, providing follow-up, treatment recommendations, and educating patients and their close contacts via health education prescriptions and offline lectures. The departments collaborated closely to improve and standardise hepatitis C screening, diagnosis, referral, and follow-up processes. The specific division of responsibilities of the cooperation group is shown in Fig. 1. A brief description of the management of patients who were positive for hepatitis C antibodies is shown in Fig. 2.

### Evaluation of the effect of the hepatitis C micro-elimination management system

#### RNA testing and treatment referral status of anti-HCV-positive patients

A retrospective study was conducted using data from January 2016 to December 2022. Anti-HCV-positive and HCV RNA-positive databases were established, comprising basic information such as patient identifiers, demographics, contact information, clinical department, and diagnosis. Duplicate values in the databases were screened using patient identifiers, and repeat visits were recorded only once. We compared the HCV RNA confirmation rate in hepatitis C antibody-positive patients and the diagnosis and treatment rates in RNA-positive patients before and after implementing the multi-disciplinary cooperation. We also analysed the characteristics of patients undergoing viral therapy.

#### Changes in the knowledge of hepatitis C among clinicians

Data on the knowledge of hepatitis C prevention and treatment among the medical staff were obtained through a ‘knowledge of management of infectious disease reporting and knowledge of diagnosis of infectious disease’ programme, which was carried out by our hospital in accordance with the requirements of the ‘Technical Guide for the Management of Infectious Disease Information Reporting in China’. The medical staff were assessed in July 2021 and March 2023, with the number of clinicians selected for the examination based on the department size, as follows: one doctor for departments with less than or equal to five clinicians, two for those with less than or equal to 10 clinicians, and three for those with more than 10 clinicians. The doctors’ number of working years and professional titles were similar to those of clinicians in the hospital. The assessment included a knowledge questionnaire on hepatitis C prevention and treatment for clinicians prepared by the Chinese Centre for Disease Control and Prevention, focusing on clinical symptoms, transmission routes, diagnostic criteria, and treatment of hepatitis C before and after the multi-disciplinary cooperation.

### Statistical analysis

Data were presented as frequencies and percentages (%). The rates were compared between the groups using the Chi-square test. The trends of the rates were tested using a Chi-square test for a linear trend. The Pearson correlation coefficient (r) was used to assess whether the correlation was positive or negative. A multivariable logistic regression was conducted to analyse factors influencing antiviral therapy in RNA-positive patients. All the tests were two-tailed, and statistical significance was set at* P* < 0.05. All analyses were performed using IBM Statistical Product and Service Solutions (SPSS), version 25. Rates were calculated as follows:

$$\mathrm{HCV}\;\mathrm{RNA}\;\mathrm{confirmation}\;\mathrm{rate}=\frac{\mathrm{Number}\;\mathrm{of}\;\mathrm{people}\;\mathrm{tested}\;\mathrm{for}\;\mathrm{HCV}\;\mathrm{RNA}}{\mathrm{Number}\;\mathrm o\;\mathrm{fpatients}\;\mathrm{who}\;\mathrm{were}\;\mathrm{anti}-\mathrm{HCV}-\mathrm{positive}\;\mathrm{in}\;\mathrm{the}\;\mathrm{same}\;\mathrm{period}}\ast100\%$$

$$\mathrm{Hepatitis}\;\mathrm C\;\mathrm{diagnosis}\;\mathrm{rate}=\frac{\mathrm{Number}\;\mathrm{of}\;\mathrm{patients}\;\mathrm{diagnosed}\;\mathrm{with}\;\mathrm{hepatitis}\;\mathrm C}{\mathrm{Number}\;\mathrm{of}\;\mathrm{patients}\;\mathrm{who}\;\mathrm{were}\;\mathrm{RNA}-\mathrm{positive}\;\mathrm{in}\;\mathrm{the}\;\mathrm{same}\;\mathrm{period}}\ast100\%$$

$$\mathrm{Hepatitis}\;\mathrm C\;\mathrm{tr}ea\mathrm{tment}\;\mathrm{rate}=\frac{\mathrm{Number}\;\mathrm{of}\;\mathrm{people}\;\mathrm{being}\;\mathrm{treated}}{\mathrm{Number}\;\mathrm{of}\;\mathrm{patients}\;\mathrm{who}\;\mathrm{were}\;\mathrm{RNA}-\mathrm{positive}\;\mathrm{in}\;\mathrm{the}\;\mathrm{same}\;\mathrm{period}}\ast100\%$$

## Results

### Demographic characteristics of the hepatitis C antibody-positive patients

Overall, 393,459 patients were screened between 2016 and 2022. Among them, 205,122 (52.133%) were male. The anti-HCV antibody-positivity rate was 0.332% (95% confidence interval [CI], 0.314%–0.350%), with 1,306 patients testing positive. Male patients had a higher HCV-positivity rate than female patients (0.372% vs. 0.287%, χ^2^ = 21.282, *P* < 0.001). The median age of anti-HCV antibody-positive patients was 54 years. Furthermore, the 25th and 75th percentiles were 39 and 65 years, respectively. The maximum age was 100 years, and the minimum age was 1 day.

**Fig. 3:**
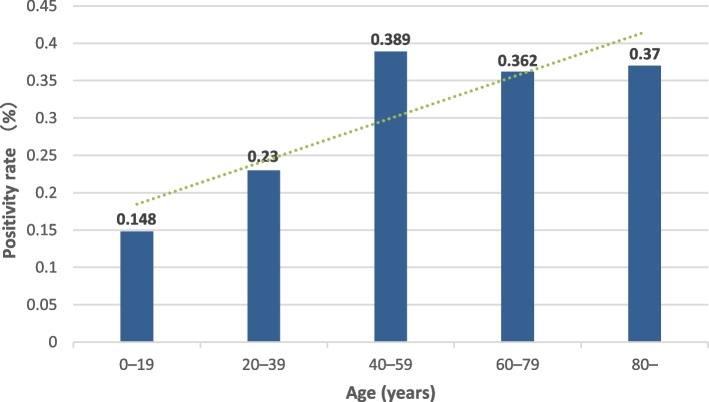
HCV-positivity rates of the different age groups

**Fig. 4:**
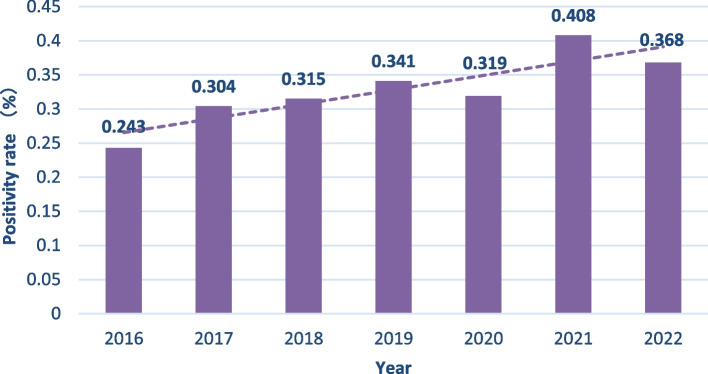
HCV-positivity rates over the different years

According to the age group, the positivity rate of anti-HCV antibodies was 0.148% among people aged 0–19 years, which was the lowest rate, while the highest rate was observed in the 40–59-year group (0.389%). Overall, 84.533% (1104/1306) of the patients positive for anti-HCV antibodies were over 40 years. The HCV antibody-positivity rate showed an increasing trend with ages (χ^2^_line_ = 45.332, *r* = 0.011, *P* < 0.001) (Fig. 3). Additionally, there was a significant increase in the anti-HCV-positivity rate, yearly from 2016 to 2022 (χ^2^_line_ = 20.417, *r* = 0.007, *P* = 0.002) (Fig. 4).

### Evaluation of the effect of the hepatitis C micro-elimination strategy

#### Changes in RNA detection, diagnosis, and treatment rates

**Fig. 5:**
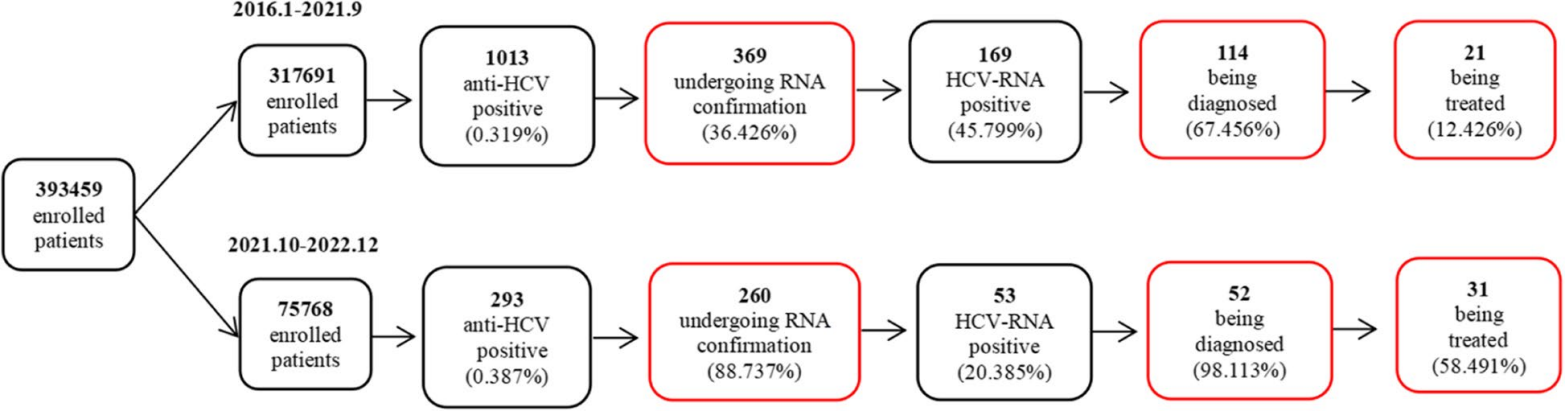
Changes in RNA confirmation, diagnosis, and treatment rates

After 2 months of preparation, the hospital launched a multi-disciplinary cooperation model in October 2021. The RNA confirmation (88.737% vs. 36.426%, *P* < 0.001), diagnosis (98.113% vs. 67.456%, *P* < 0.001), and treatment rates (58.491% vs. 12.426%, *P* < 0.001) were significantly improved compared with those before the intervention (Fig. 5).

Each patient’s treatment status was assessed by viewing their medical records and telephone follow-up information. Based on this information, the multivariable logistic regression analysis identified age > 40 years (40–59 years: odds ratio (OR): 9.135, 95%CI: 1.815–45.986; ≥ 60 years: OR: 9.999, 95%CI: 2.878–34.737), living in other cities (OR: 3.641, 95%CI: 1.346–9.851), urban resident insurance (OR: 3.742, 95%CI: 1.388–10.090), and tumours or other serious diseases (OR: 5.533, 95%CI: 1.597–19.172) as adverse factors for antiviral treatment. Conversely, the multi-disciplinary cooperation model (OR: 0.024, 95%CI: 0.007–0.074) and the presence of hepatic fibrosis (OR: 0.109, 95%CI: 0.034–0.352) were favourable factors for antiviral therapy (Table 1). Of the patients receiving antiviral therapy, 23.077% (12/52) were in the course of treatment, 25% (13/52) had completed treatment but were not followed up for 6 months (24 weeks), and 48.077% (25/52) had completed treatment and the 6-month (24 weeks) follow-up and achieved an SVR. Overall, 0.385% (2/52) of patients died of other primary diseases.

**Table 1:** Characteristics of the RNA-positive individuals who underwent antiviral therapy

Variable	Antiviral therapy (52)		No antiviral therapy (170)		χ^2^	Univariate logistic regression analysis OR (95%*CI*)	Multivariable logistic regression analysis OR (95%*CI*)
	**Number**	**Rate (%)**	**Number**	**Rate (%)**	***P*-value**		
**Age(years)**							
< 40	8	36.364	14	63.636	10.453	Ref	Ref
40–59	31	30.392	71	69.608	0.005	2.855 (1.389–5.866)	9.135 (1.815–45.986)
≥ 60	13	13.265	85	86.735		3.736 (1.312–10.640)	9.999 (2.878–34.737)
**Sex**							
Male	33	23.239	109	76.761	0.007	Ref	Ref
Female	19	23.75	61	76.25	0.931	0.972 (0.510–1.854)	0.807 (0.329–1.980)
**Residence**							
Fuzhou	36	29.752	85	70.248	5.939	Ref	Ref
Other cities	16	15.842	85	84.158	0.015	2.250 (1.162–4.359)	3.641 (1.346–9.851)
**Insurance**							
Employee health insurance	39	28.676	97	71.324	5.401	Ref	Ref
Urban residents’ basic medical insurance	13	15.116	73	84.884	0.020	2.258 (1.124–4.534)	3.742 (1.388–10.090)
**HCV multi-disciplinary cooperation in the hospital**							
2016.1–2019.9	21	12.426	148	87.574	47.731	Ref	Ref
2019.10–2022.12	31	58.491	22	41.509	< 0.001	0.101 (0.049–0.205)	0.024 (0.007–0.074)
**Hepatic status**							
No fibrosis	37	21.143	138	78.857	2.397	Ref	Ref
Hepatic fibrosis	15	31.915	32	68.085	0.122	0.572 (0.280–1.166)	0.109 (0.034–0.352)
**Tumours or other serious diseases**							
Without	42	27.451	111	72.549	4.452	Ref	Ref
With	10	14.492	59	85.507	0.035	2.232 (1.046–4.766)	5.533 (1.597–19.172)

Tumours or other serious diseases refer to conditions such as oesophageal cancer, gastric cancer, haemophilia, and syringomyelia

*HCV* hepatitis C virus

#### Changes in HCV-related knowledge among clinicians

In 2021 and 2023, 158 and 159 clinicians, respectively, were included in the examination. There were no statistically significant differences in age, education level, or professional title among the clinicians between 2021 and 2023 (*P* > 0.05). However, significant improvements were observed in relation to the clinicians’ knowledge, including their ability to understand hepatitis C characteristics (93.711% vs. 58.861%, *P* < 0.001), identify high-risk individuals (94.340% vs. 53.797%, *P* < 0.001), manage the process after diagnosis (88.679% vs. 67.089%, *P* < 0.001), and effectively perform treatment (84.277% vs. 51.899%, *P* < 0.001). Specifically, before the implementation of the multi-disciplinary cooperation model, 27.215% of the clinicians stated that hepatitis C infection could be prevented by vaccine injection, and 18.987% of the clinicians believed that most of the patients with hepatitis C had obvious clinical symptoms. Furthermore, 20.253% of the clinicians noted that those who shared tableware with hepatitis C-infected persons were at high risk of HCV infection. Approximately 32.911% of the clinicians believed that hepatitis C-infected patients who were asymptomatic did not require treatment, and 44.304% stated that interferon and ribavirin were the most effective drugs for the treatment of hepatitis C. These misconceptions were diminished significantly after implementing the multi-disciplinary cooperation model (Table 2).

**Table 2:** Changes in HCV-related knowledge among clinicians

Questions (Answers)	2021 (158)		2023 (159)		χ^2^
	**Number**	**Rate (%)**	**Number**	**Rate (%)**	***P*-value**
1. **What are the characteristics of hepatitis C? (B and C)**	93	58.861	149	93.711	53.289
A. Most people have obvious symptoms	30	18.987	5	3.145	
B. Develops into chronic hepatitis easily	145	91.772	159	100	< 0.001
C. Develops into liver cirrhosis and cancer	153	96.835	158	99.371	
D. Vaccination can prevent hepatitis C infection	43	27.215	10	6.289	
E. Unknown	1	0.633	0	0	
**2. What are the transmission routes of hepatitis C? (A, C, and D)**	139	87.975	151	94.969	4.975
A. Blood-borne transmission	153	96.835	159	100	
B. Respiratory transmission	5	3.165	1	0.629	0.026
C. Sexual transmission	150	94.937	153	96.226	
D. Maternal-neonatal transmission	153	96.835	159	100	
E. Transmission through the digestive tract	10	6.329	1	0.629	
F. Unknown	1	0.6333	0	0	
**3. Who are the people at high risk for HCV infection? (A, B, C, D, F, G, and H)**	85	53.797	150	94.340	67.927
A. Those with a history of intravenous drug addiction	153	96.835	159	100	
B. Paid blood donors	129	81.646	159	100	< 0.001
C. Multiple sexual companions	147	93.038	159	100	
D. Men who have sex with men	137	86.709	156	98.113	
E. Those sharing tableware with patients with hepatitis C	32	20.253	6	3.774	
F. Those with exposure of mucous membranes to contaminated blood	153	96.835	159	100	
G. Those with tattoos and pierced ears	150	94.937	152	95.597	
H. Those with a history of iatrogenic exposure such as surgery and dialysis	152	96.203	157	98.742	
J. Unknown	0	0	0	0	
**4. What is the basis of diagnosis for confirmed cases of hepatitis C? (A)**	145	91.772	158	99.371	10.84
A. HCV RNA-positive	145	91.772	158	99.371	
B. Anti-HCV-positive	9	5.696	1	0.629	0.001
C. None of the above	2	1.266	0	0	
D. Unknown	2	1.266	0	0	
**5. What period of infection is defined as chronic HCV infection? (B)**	133	84.177	153	96.226	13.041
A. Persistent infection exceeding three months	17	10.759	3	1.887	
B. Persistent infection exceeding six months	133	84.177	153	96.226	< 0.001
C. Persistent infection exceeding 12 months	7	4.430	3	1.887	
D. Unknown	1	0.633	0	0	
**6. What are the processes for the management of patients with hepatitis C after the diagnosis?** **(A, B, and D)**	106	67.089	141	88.679	21.471
A. Reporting of infectious diseases	153	96.835	159	100	
B. Clinically diagnosed cases require HCV RNA testing	153	96.835	159	100	< 0.001
C. Do not intervene unless there is any discomfort	52	32.911	15	9.434	
D. Refer cases to the liver clinic for antiviral therapy	133	84.177	159	100	
E. Unknown	0	0	0	0	
**7. What is the most effective treatment for hepatitis C? (D)**	82	51.899	134	84.277	38.268
A. Reduce liver enzyme levels	3	1.899	0	0	
B. Traditional Chinese treatment	0	0	0	0	< 0.001
C. Ribavirin and interferon	70	44.304	25	15.723	
D. DAAs	82	51.899	134	84.277	
E. Unknown	2	1.266	0	0	
**8. Has the country included drugs for HCV treatment in the medical insurance? (A)**	146	92.405	159	100	12.551
A. Yes	146	92.405	159	100	
B. No	9	5.696	0	0	< 0.001
C. Unknown	3	1.899	0	0	

*HCV* hepatitis C virus, *DAAs* direct-acting antiviral agents

## Discussion

### Changes before and after implementing the multi-disciplinary cooperation model

This retrospective analysis of hepatitis C serology revealed that among 1,306 anti-HCV-positive cases, more men than women tested positive, potentially attributable to the higher trends of unsafe exposure behaviour in men [21]. Similar findings were reported in other studies [10, 19]. Furthermore, 84.533% of the patients were aged ≥ 40 years, highlighting the need for increased anti-HCV screening in male patients ≥ 40 years [22, 23]. Our findings also showed an annual increase in the anti-HCV antibody-positivity rate, consistent with the observations from previous research [24, 25]. However, hepatitis C elimination has been delayed owing to the impact of COVID-19, and national HCV surveillance systems are still being established. Only 18% of patients with hepatitis C in China had been diagnosed correctly. Although 25% of patients were in need of urgent treatment, only 1.3% had access to treatment [26].

Before the multi-disciplinary cooperation, doctors at this hospital had limited awareness of hepatitis C, and only 51.899% of the clinicians recognised DAAs as the preferred treatment, while approximately 32.911% believed that asymptomatic patients did not require treatment. The professional experience and perception regarding hepatitis C among physicians can affect the medical behaviour and willingness of patients to receive treatment significantly [27, 28]. In this study, we found that before the implementation, 36.426% of the anti-HCV-positive patients had undergone confirmatory HCV RNA testing, 67.456% of the RNA-positive patients were diagnosed correctly with hepatitis C, and only 12.426% of the RNA-positive patients received antiviral therapy. Overall, referral diagnosis and treatment rates were low, as observed in other countries [29].

Under the multi-disciplinary cooperation, public health doctors supervised the process, by using the infectious disease reporting and management platform to track RNA detection in patients with anti-HCV positivity, communicate with the doctor in charge, and ensure timely HCV RNA testing. Active follow-ups are recommended through SMS or phone calls, to spread awareness about treatment and prevention and ensure that new patients with hepatitis C in the hospital complete their treatment course. This study showed that 88.737% of patients with anti-HCV positivity were prescribed an HCV RNA test, 98.113% of the RNA-positive patients were diagnosed with hepatitis C, and 58.491% were referred for treatment.

However, the treatment rate still fell short of the target of 80% of hepatitis C patients receiving treatment. In this regard, multivariable logistic regression analysis revealed adverse factors for HCV antiviral therapy, including age ≥ 40 years, non-local residency; urban resident insurance; lack of intervention by medical institutions; and absence of liver fibrosis or tumours or other serious diseases (including oesophageal cancer, gastric cancer, haemophilia, and syringomyelia). Among these factors, the OR for multi-disciplinary cooperation in medical institutions showed the strongest correlation and it was the most important factor for patients to undergo HCV treatment. Implementing multi-disciplinary cooperation in all healthcare settings can facilitate the elimination of hepatitis C.

### Efforts to eliminate hepatitis C in China

To eliminate hepatitis C by 2030, China must treat at least 550,000 cases annually [30]. Since 2017, an increasing number of DAAs have been approved [31]. Currently, eight DAAs are included in the national medical insurance directory, which has greatly reduced the economic burden on patients with hepatitis C. For example, regarding sofosbuvir/velpatasvir (SOF/VEL), a pan-genotypic drug, before including the drug in the national medical insurance drug negotiations, the cost of a full treatment course was approximately $12,000. Currently, the drug cost is approximately $1500. After including SOF/VEL in the medical insurance negotiations, the maximum cost of the full treatment course was not more than $900 under the urban resident basic medical insurance, while it was $600 under the employee health insurance, which improved drug accessibility. Our study showed that employee health insurance was a favourable factor for antiviral therapy in patients with HCV because it requires a lower percentage of out-of-pocket expenses.

In September 2021, the National Health Commission of China, the Ministry of Science and Technology, and nine other departments formulated and issued the Work Plan for Action to Eliminate the Public Health Hazards of Hepatitis C (2021–2030) [32]. Thus, the government aims to implement effective measures to significantly prevent and control hepatitis C, curb new infections, effectively detect and cure patients, and reduce mortality from liver cirrhosis and cancer caused by hepatitis C. The Work Plan also proposed specific action targets for 2021, 2025, and 2030.

### Problems in eliminating hepatitis C in current medical institutions

Although we improved the hepatitis C diagnosis rate, the treatment rate remains suboptimal. First, DAAs are not always available at many medical institutions. A study by the Chinese Centre for Disease Control and Prevention found that only 10.84% of medical institutions followed the standard treatment guidelines for hepatitis C in 2020 [33]. Our study showed lower treatment rates for patients from other cities, possibly because of the limited access to treatment in their city. The lack of DAAs drugs in hospital facilities is an important factor. Under the diagnosis-related group/diagnosis-intervention packet payment reform, the health insurance fund will make settlements according to the established payment standard. The medical institutions will bear the excess medical expenses over the base payment. If there is a surplus, it can be used as income for the hospital [34]. Therefore, the high cost of the negotiated drugs poses cost control challenges, further reducing motivation to introduce DAAs. On April 22, 2021, the National Healthcare Administration and the National Health Commission jointly issued the Guidance on Establishing and Improving the Dual-Channel Management Mechanism for negotiated drugs under the national medical insurance jointly [35]. Consequently, after clinicians at the hospital issue the prescriptions, patients can purchase and pay for the relevant drugs in the designated pharmacy covered by the medical insurance, without a proportional increase in drug costs contributing to the total health expenditure. However, to date, the policy has not been well implemented, and most hospitals still do not operate a dual-channel mechanism.

Second, only 46.1% of the medical institutions in China are able to conduct RNA testing [33]. Furthermore, hospitals capable of RNA testing accumulate samples for cost efficiency, impacting the accuracy of the RNA results. Simultaneously, if the RNA test results are released after the patients are discharged, treatment initiation will not be conducive, particularly for non-local patients, which can affect the treatment rate.

Third, China only began to include DAAs in the Category B list of medical insurance in 2019, and the treatment is not yet universal. Currently, clinicians may not fully understand the clinical indications for hepatitis C antiviral drugs, especially the impact of DAAs on patients with hepatitis C combined with tumours or other serious diseases. The follow-up results of the patients who tested positive for HCV RNA revealed that the treatment rate was low for those aged ≥ 60 years or with tumours or other serious diseases. However, some patients and doctors may decline the hepatitis C treatment due to a lack of awareness regarding the types of medication and importance of treating hepatitis C.

### Limitations

This study had several limitations. First, this was a retrospective study that was conducted in a single centre that included experience sharing, despite our institution being a large, regional, comprehensive grade-A hospital that treats patients with various diseases. However, the findings may reflect the real-world situation of hepatitis C in the region. Future studies could use multiple, decentralised diagnostic care pathways to improve the detection of potential infections [36]. This may help us better understand the local prevalence of hepatitis C. Second, treatment information was primarily obtained from medical records and via telephone follow-up interviews. Currently, a transformation of the treatment and follow-up data platform has been initiated at our hospital. In the future, clinicians will be able to directly input relevant information to improve the accuracy of treatment-related data. Finally, there was still a percentage of anti-HCV( +) patients who did not receive HCV RNA tests. The gap in accurate HCV RNA diagnosis requires reduction; however, due to the layout of the hospital districts, we could not perform a reflex testing model, such as that built by Huang et al. [37]. Future studies should be performed in centres which allow for rapid blood sample transfer from collection to testing for such analysis.

## Conclusions

China has the highest number of HCV infections worldwide and is far from reaching the goal of eliminating hepatitis C [38]. To the best of our knowledge, this is the first study to evaluate the hepatitis C micro-elimination strategy in a hospital in China. The micro-elimination strategy enables timely referrals and standardised treatment for more patients by establishing multi-sectoral collaborative screening and referral paths, which play an important role in actual clinical situations. Moreover, we anticipate additional government interventions to ensure drug supply and improved RNA testing for HCV. Furthermore, ongoing Chinese pharmacoeconomic research on hepatitis C in different populations can provide valuable data supporting the use of DAAs. These collective efforts will help accelerate the progress eliminating hepatitis C in China.

## Data Availability

The raw data supporting the conclusions of this article will be made available by the corresponding author upon reasonable request.

## Abbreviations

OR: Odds ratio
HCV: Hepatitis C virus
DAAs: Direct-acting antiviral agents
WHO: World Health Organization
SVR: Sustained virologic response
COVID-19: Coronavirus disease 2019
SMS: Short message service
R: Pearson correlation coefficient
CI: Confidence interval

## References

[1] World Health Organization. Hepatitis C. 2022. Available at: https://www.who.int/news-room/fact-sheets/detail/hepatitis-c. Accessed 12 May 2023.

[2] Irshad M, Mankotia DS, Irshad K (2013). An insight into the diagnosis and pathogenesis of hepatitis C virus infection. World J Gastroentero.

[3] Chinese Society of Hepatology, Chinese Society of Infectious Diseases, Chinese Medical Association (2019). Guidelines for the prevention and treatment of hepatitis C (2019 version). Zhonghua Gan Zang Bing Za Zhi..

[4] Cooke GS, Andrieux-Meyer I, Applegate TL, Atun R, Burry JR, Cheinquer H, Dusheiko G, Feld JJ, Gore C, Griswold MG (2019). Accelerating the elimination of viral hepatitis: a Lancet Gastroenterology & Hepatology Commission. Lancet Gastroenterol Hepatol.

[5] World Health Organization. Guidelines for the care and treatment of persons diagnosed with chronic hepatitis C virus infection. World Health Organization; 2018. https://iris.who.int/handle/10665/273174. 30307724

[6] World Health Organization. Combating hepatitis B and C to reach elimination by 2030: advocacy brief. World Health Organization. https://apps.who.int/iris/handle/10665/206453.

[7] World Health Organization. Global hepatitis report 2017. Available at: https://www.who.int/publications/i/item/9789241565455. Accessed 13 May 2023.

[8] Polaris Observatory HCV Collaborators (2022). Global change in hepatitis C virus prevalence and cascade of care between 2015 and 2020: a modelling study. Lancet Gastroenterol Hepatol.

[9] Chinese Society of Hepatology, Chinese Society of Infectious Diseases, Chinese Medical Association (2022). Guidelines for the prevention and treatment of hepatitis C (2022 version). Zhonghua Gan Zang Bing Za Zhi..

[10] Hei FX, Ye SD, Ding GW, Pang L, Wang XC, Liu ZF (2018). Epidemiological analysis on reported hepatitis c cases in China from 2012 to 2016. Biomed Environ Sci.

[11] National Health and Family Planning Commission, National Development and Reform Commission, Ministry of Education Ministry of Science and Technology, Ministry of Industry and Information Technology, Ministry of Public Security (2018). China viral hepatitis prevention and control plan (2017–2020). Chin J Viral Dis..

[12] Blach S, Kondili LA, Aghemo A, Cai Z, Dugan E, Estes C, Gamkrelidze I, Ma S, Pawlotsky JM, Razavi-Shearer D (2021). Impact of COVID-19 on global HCV elimination efforts. J Hepatol.

[13] Tergast TL, Blach S, Tacke F, Berg T, Cornberg M, Kautz A, Manns M, Razavi H, Sarrazin C, Serfert Y (2022). Updated epidemiology of hepatitis C virus infections and implications for hepatitis C virus elimination in Germany. J Viral Hepat.

[14] Winter RJ, Holmes JA, Papaluca TJ, Thompson AJ (2022). The importance of prisons in achieving hepatitis C elimination: insights from the Australian experience. Viruses-Basel.

[15] McLeod A, Hutchinson SJ, Smith S, Leen C, Clifford S, McAuley A, Wallace LA, Barclay ST, Bramley P, Dillon JF (2021). Increased case-finding and uptake of direct-acting antiviral treatment essential for micro-elimination of hepatitis C among people living with HIV: a national record linkage study. HIV Med.

[16] Rajasekaran A, Franco RA, Overton ET, McGuire BM, Towns GC, Locke JE, Sawinski DL, Bell EK (2021). Updated pathway to micro-elimination of hepatitis C virus in the hemodialysis population. Kidney Int Rep.

[17] Lazarus JV, Wiktor S, Colombo M, Thursz M, EASL International Liver Foundation (2017). Micro-elimination - A path to global elimination of hepatitis C. J Hepatol..

[18] Lazarus JV, Safreed-Harmon K, Thursz MR, Dillon JF, El-Sayed MH, Elsharkawy AM, Hatzakis A, Jadoul M, Prestileo T, Razavi H (2018). The micro-elimination approach to eliminating hepatitis C: strategic and operational considerations. Semin Liver Dis.

[19] Liu L, Xu H, Hu Y, Shang J, Jiang J, Yu L, Zhao C, Zhang D, Zhang X, Li J (2019). Hepatitis C screening in hospitals: find the missing patients. Virol J.

[20] Chen CJ, Huang YH, Hsu CW, Chen YC, Chang ML, Lin CY, Shen YH, Chien RN (2023). Hepatitis C micro-elimination through the retrieval strategy of patients lost to follow-up. BMC Gastroenterol.

[21] He N, Hao S, Feng G, Gao J, Kong FJ, Ren ZX, Xu MQ, Yang YQ (2021). Analysis of the factors influencing the elimination strategies with the current status of diagnosis and treatment of hepatitis C in hospital. Zhonghua Gan Zang Bing Za Zhi..

[22] Xu R, Yu Y, Leitch ECM, Wang M, Huang K, Huang J, Tang X, Liao Q, Song D, Shan Z (2019). HCV genotype 6 prevalence, spontaneous clearance and diversity among elderly members of the Li ethnic minority in Baisha County. China J Viral Hepat.

[23] Li Y, Zhao L, Geng N, Zhu W, Liu H, Bai H (2020). Prevalence and characteristics of hepatitis C virus infection in Shenyang City, Northeast China, and prediction of HCV RNA positivity according to serum anti-HCV level: retrospective review of hospital data. Virol J.

[24] Zhao Z, Chu M, Guo Y, Yang S, Abudurusuli G, Frutos R, Chen T (2022). Feasibility of hepatitis C elimination in China: From epidemiology, natural history, and intervention perspectives. Front Microbiol.

[25] Yang J, Qi JL, Wang XX, Li XH, Jin R, Liu BY, Liu HX, Rao HY (2023). The burden of hepatitis C virus in the world, China, India, and the United States from 1990 to 2019. Front Public Health.

[26] Hui Z (2017). Report on the Status of Hepatitis C Infection in China and Prevention and Treatment Strategies.

[27] Joukar F, Mansour-Ghanaei F, Soati F, Meskinkhoda P (2012). Knowledge levels and attitudes of health care professionals toward patients with hepatitis C infection. World J Gastroenterol.

[28] Korkmaz P, Uyar C, Ozmen A, Toka O (2016). Knowledge and attitude of health care workers toward patients with hepatitis C infection. Southeast Asian J Trop Med Public Health.

[29] Yehia BR, Schranz AJ, Umscheid CA, Lo Re V (2014). The treatment cascade for chronic hepatitis C virus infection in the United States: a systematic review and meta-analysis. PLoS One..

[30] Wei L (2018). Chronic hepatitis C: achievement, challenge and strategy to eliminate as a public health threat. Zhonghua Gan Zang Bing Za Zhi..

[31] Li M, Zhuang H, Wei L (2019). How would China achieve WHO's target of eliminating HCV by 2030?. Expert Rev Anti Infect Ther.

[32] Li J, Pang L, Liu Z (2022). Interpretation of the National Action Plan for Eliminating Hepatitis C as a Public Health Threat (2021–2030). China CDC Weekly.

[33] Feng X, Ding G, Yu H, Pan L, Gao Y, Tang Y, Mao Y, Lin P (2020). Current status of hepatitis C diagnosis and treatment in Chinese medical institutions. Chin J AIDS STD..

[34] Zhao C, Wang C, Shen C, Wang Q (2018). Diagnosis-related group (DRG)-based case-mix funding system, a promising alternative for fee for service payment in China. Biosci Trends.

[35] Fu R, Mao N (2023). Analysis on the circulation and obstacles of innovative drugs in retail terminal under the dual-channel policy in China. Health Economics Research..

[36] Robinson E, Byrne CJ, Carberry J, Radley A, Beer LJ, Inglis SK, Tait J, Macpherson I, Goldberg D, Hutchinson SJ (2023). Laying the foundations for hepatitis C elimination: evaluating the development and contribution of community care pathways to diagnostic efforts. BMC Public Health.

[37] Huang CF, Wu PF, Yeh ML, Huang CI, Liang PC, Hsu CT, Hsu PY, Liu HY, Huang YC, Lin ZY (2021). Scaling up the in-hospital hepatitis C virus care cascade in Taiwan. Clin Mol Hepatol.

[38] Bian DD, Zhou HY, Liu S, Liu M, Duan C, Zhang JY, Jiang YY, Wang T, Chen Y, Wang Z (2017). Current treatment status and barriers for patients with chronic HCV infection in mainland China: a national multicentre cross-sectional survey in 56 hospitals. Medicine (Baltimore).

